# Knockdown of lncRNA PVT1 alleviates high glucose-induced proliferation and fibrosis in human mesangial cells by miR-23b-3p/WT1 axis

**DOI:** 10.1186/s13098-020-00539-x

**Published:** 2020-04-15

**Authors:** Wen Zhong, Jiaoe Zeng, Junli Xue, Aimin Du, Yancheng Xu

**Affiliations:** 1 Department of Endocrinology, Zhongnan Hospital of Wuhan University, 169 East Lake Road, Wuchang District, Wuhan, 430071 Hubei China; 2 Department of Endocrine, Jingzhou Central Hospital and The Second Clinical Medical College, Yangtze University, Jingzhou, 434020 Hubei China

**Keywords:** DN, PVT1, miR-23b-3p, WT1, High glucose

## Abstract

**Background:**

Diabetic nephropathy (DN) is a severe complication of diabetes with type 1 and 2. Long non-coding RNAs (lncRNAs) are being found to be involved in the DN pathogenesis. In this study, we aimed to further explore the effect and underlying mechanism of plasmacytoma variant translocation 1 (PVT1) in DN pathogenesis.

**Methods:**

The expression levels of PVT1, miR-23b-3p, and Wilms tumor protein 1 (WT1) mRNA were assessed by quantitative real-time polymerase chain reaction (qRT-PCR). Western blot analysis was performed to determine protein expression. Cell proliferation was detected using the 3-(4,5-dimethylthiazol-2-yl)-5-(3-carboxymethoxyphenyl)-2-(4-sulfophenyl)-2*H*-tetr-azolium (MTS) assay. The targeted correlation between miR-23b-3p and PVT1 or WT1 was verified by dual-luciferase reporter assay.

**Results:**

PVT1 and WT1 were highly expressed in the serum of DN patients and high glucose (HG)-induced mesangial cells (MCs). The knockdown of PVT1 or WT1 ameliorated HG-induced proliferation and fibrosis in MCs. Mechanistically, PVT1 modulated WT1 expression through acting as a molecular sponge of miR-23b-3p. The miR-23b-3p/WT1 axis mediated the protective effect of PVT1 knockdown on HG-induced proliferation and fibrosis in MCs. The NF-κB pathway was involved in the regulatory network of the PVT1/miR-23b-3p/WT1 axis in HG-induced MCs.

**Conclusion:**

Our study suggested that PVT1 knockdown ameliorated HG-induced proliferation and fibrosis in MCs at least partially by regulating the miR-23b-3p/WT1/NF-κB pathway. Targeting PVT1 might be a potential therapeutic strategy for DN treatment.

## Highlights

1. The knockdown of PVT1 or WT1 ameliorated HG-induced MCs proliferation and fibrosis.
2. PVT1 modulated WT1 expression through acting as a molecular sponge of miR-23b-3p.
3. PVT1 knockdown ameliorated HG-induced proliferation and fibrosis in MCs possibly through the miR-23b-3p/WT1 axis.

## Background

Diabetic nephropathy (DN) is a severe complication of type 1 and 2 diabetes all over the world [1, 2]. DN is histologically characterized by mesangial expansion, tubulointerstitial fibrosis and glomerulosclerosis, which are induced by the hyper-proliferation of mesangial cells (MCs) and the accumulation of extracellular matrix (ECM) proteins [3, 4]. Although DN pathogenesis is complicated and ambiguous, hyperglycemia has been identified to contribute to the development and progression of this disease [2]. Despite significant advances in DN management, effective strategies against DN remain limited. Therefore, it is very imperative to develop new therapeutic targets for DN treatment.

Long non-coding RNAs (lncRNAs) are a diverse family of > 200 nucleotides RNA transcripts [5]. LncRNAs serve as important regulators in physiopathology processes via various mechanisms, including their microRNA (miRNAs) binding functions [6]. Emerging researches have suggested that lncRNAs are implicated in the pathogenesis and development of DN [7–9]. Plasmacytoma variant translocation 1 (PVT1), a 1.9 kb long lncRNA, has been found as a potential locus for diabetic end-stage kidney disease (ESRD) by a pooling-based genome polymorphism study [10]. It was reported that PVT1 was up-regulated in human podocytes and mouse podocyte clone 5 podocytes after high glucose (HG) exposure and PVT1 knockdown attenuated HG-induced damage and apoptosis in podocytes [11]. Moreover, PVT1 expression was elevated in HG-induced human MCs, and its depletion weakened the expression of several ECM proteins, eliciting a crucial involvement of PVT1 in DN pathogenesis [12]. In the current research, we aimed was to further explore the effect and underlying mechanism of PVT1 on HG-induced MCs proliferation and fibrosis.

Wilms tumor protein 1 (WT1) is a zinc finger-like transcription factor, which is pivotal for renal development and closely associated with tumorigenesis in the kidney [13]. WT1 has been identified to be up-regulated in diabetic patients with proteinuria, and patients with WT1-positive urinary exosomes have reduced renal function [14]. Emerging evidence has shown that urinary exosomal WT1 functions as a podocyte-specific marker for DN diagnosis and prognosis [15, 16]. The networks of the competing endogenous RNAs (ceRNAs) have recently been reported to play crucial roles in DN pathogenesis [17, 18]. Two putative targeted correlations between miR-23b-3p and PVT1 or WT1 were predicted by the database of the online software algorithms, eliciting a potential ceRNA network of the PVT1/miR-23b-3p/WT1 axis in DN progression. In this study, we focused on the role and underlying mechanism of PVT1 in HG-induced MCs. Furthermore, our study suggested that PVT1 knockdown alleviated HG-induced proliferation and fibrosis in human MCs possibly through targeting the miR-23b-3p/WT1/nuclear factor-κB (NF-κB) pathway.

## Materials and methods

### Clinical specimens and ethics statement

34 serum samples were obtained from type 2 diabetic patients with clinical or biopsy confirmed DN, from Zhongnan Hospital of Wuhan University. A control sample set was obtained by the recruitment of 30 healthy volunteers, who have not suffered from DN, diabetes, inflammatory disease, or autoimmune diseases. Serum samples were stored at − 80 °C. Each participant signed written informed consent before sample collection. Our study was approved by the Ethics Committee of Zhongnan Hospital of Wuhan University.

### Cell culture and treatment

Human MCs (Cell Bank of Chinese Academy of Sciences, Shanghai, China) was grown in Dulbecco’s modified Eagle medium (DMEM; Invitrogen, Darmstadt, Germany) with 10% fetal calf serum (FCS, Australian Biosearch, Karrinyup, Australia) and 1% antibiotics (Invitrogen) at 37 °C, 5% CO_2_. For HG treatment, MCs were cultured in medium containing 45 nM of d-glucose (Amresco, Solon, OH, USA) for 48 h.

### Oligonucleotide and plasmid transfection

For PVT1 or WT1 silencing, small-interfering RNA (siRNA) against PVT1 (si-PVT1#1 and si-PVT1#2) or WT1 (si-WT#1 and si-WT#2) was transfected into MCs, and a nontarget siRNA was employed as the negative control. For miR-23b-3p alteration, the synthetic miR-23b-3p mimic or its inhibitor (anti-miR-23b-3p) was introduced into MCs, with a corresponding scrambled sequence (miR-NC mimic or anti-miR-NC) as the negative control. PVT1 or WT1 up-regulation was performed using PVT1 or WT1 overexpression plasmid, with a nontarget pcDNA3.1 vector (pcDNA) as a negative control. All oligonucleotides and plasmids were bought from GenePharma (Shanghai, China). The commercial Lipofectamine 3000 transfection reagent (Invitrogen) was used for each transfection.

### Quantitative real-time polymerase chain reaction (qRT-PCR)

Total RNA from serum samples and MCs was prepared by using TRIzol reagent (Invitrogen). Reverse transcription PCR was carried out using SuperScript™ III reverse transcriptase (Invitrogen) for PVT1 and WT1 and TaqMan MicroRNA Reverse Transcription kit (Applied Biosystems, Tokyo, Japan) for miR-23b-3p. qRT-PCR was implemented using the Universal SYBR Green Master kit (Roche, Tokyo, Japan) for PVT1 and WT1 and TaqMan MicroRNA assay kit (Applied Biosystems) for miR-23b-3p. The expression levels of PVT1, WT1, and miR-23b-3p were normalized to β-actin or U6 snRNA, and calculated by 2^−ΔΔCt^ cycle threshold method. The primers (5′–3′) used in the experiments were as follows: PVT1-forward, TGAGAACTGTCCTTACGTGACC and PVT1-reverse, AGAGCACCAAGACTGGCTCT; WT1-forward, AGGGTACGAGAGCGATAACCACAC and WT1-reverse, TCAGATGCCGACCGTACAAGA; β-actin-forward, GCACCACACCTTCTACAATG and β-actin-reverse, TGCTTGCTGATCCACATCTG; miR-23b-3p-forward, GAGCATCACATTGCCAGGG and miR-23b-3p-reverse, CATCCGTAAAGACCTCTATGCCAAC; U6 snRNA-forward, CTCGCTTCGGCAGCACA and U6 snRNA-reverse, AACGCTTCACGAATTTGCGT.

### Western blot

Total protein was extracted, separated, and transferred onto polyvinylidene difluoride (PVDF, Dupont NEN, Boston, MA, USA) membranes, as described previously [19]. The membranes were probed with the following primary antibodies: anti-WT1 (ab89901, 1:1000), anti-Ki67 (ab92742, 1:5000), anti-p65 (ab16502, 1:1000), anti-phosphorylated p65 (anti-p-p65, ab86299, 1:5000), anti-α-smooth muscle actin (anti-α-SMA, ab32575, 1:2000), anti-fibronection (anti-FN, ab34219, 1:1000) and anti-glyceraldehyde 3-phosphate dehydrogenase (anti-GAPDH, ab181602, 1:10,000). The goat anti-mouse or anti-rabbit horseradish peroxidase (HRP)-conjugated IgG antibody (ab6728 or ab6721, 1:5000) was used as the secondary antibody. Protein blot was developed using the enhanced chemiluminescence (ECL) detection kit (Pierce Biotechnology, Bonn, Germany) and analyzed by the ChemiDoc™ XRS imaging system (Bio-Rad, Tokyo, Japan) with Image Lab™ software. All antibodies used for western blot analysis were purchased from Abcam (Cambridge, UK).

### Cell proliferation assay

The proliferation ability of MCs after HG treatment or the indicated transfection was monitored using the colorimetric 3-(4,5-dimethylthiazol-2-yl)-5-(3-carboxymethoxyphenyl)-2-(4-sulfophenyl)-2*H*-tetr-azolium (MTS) assay. Briefly, MCs (1.0 × 10^6^/well) grown in 96-well plates were subjected to the indicated various treatments. After 0, 1, 2, and 3 days, 20 µL of MTS/phenazine methosulfate (PMS) reagent (Promega, Leiden, The Netherlands) was used and incubated for 3–4 h at 37 °C. The quantify of formazan was proportional to the number of viable cells which could be determined by a CellTiter 96^®^ AQueous One solution cell proliferation assay system (Promega).

### Bioinformatics and dual-luciferase reporter assay

The targeted miRNAs of PVT1 and the molecular targets of miR-23b-3p were predicted using the online software miRcode (http://www.mircode.org/) and TargetScan Human 7.1 (http://www.targetscan.org/vert_71/), respectively. PVT1 and WT1 3′-UTR reporter constructs (PVT1-WT and WT1-WT) harboring the complementary sequence of miR-23b-3p, and the site-directed mutant of the target sequence (PVT1-MUT and WT1-MUT) were obtained from Hanbio (Shanghai, China). The reporter vectors were introduced into MCs, respectively, together with miR-23b-3p mimic or miR-NC mimic. Firefly and Renilla luciferase activities were simultaneously detected by the Dual-luciferase Reporter Assay System (Promega) after 48 h transfection. Relative luciferase activity was normalized against Renilla luciferase activities and presented as percentage of suppression.

### RNA pulldown and RNA immunoprecipitation (RIP) assays

Cell lysates of MCs were prepared using the Complete Lysis-M reagent (Roche Diagnostic, Basel, Switzerland) following the instructions of producers. In RNA pulldown assays, the lysates were incubated with biotin-labeled miR-23b-3p mimic (bio-miR-23b-3p-WT, Ribobio, Guangzhou, China), its mutant in the seed sequence (bio-miR-23b-3p-MUT, Ribobio) or the negative control (bio-NC, Ribobio) at 4 °C for 4 h before adding to the Streptavidin agarose beads (Sigma-Aldrich, Sternheim, Germany) for 2 h. In RIP assays, cell lysates were incubated with magnetic beads-conjugated anti-Argonaute2 (anti-Ago2, Abcam) or anti-IgG (Abcam) antibody at 4 °C for 4 h. In both assays, the beads were harvested, and total RNA was extracted for the assessment of PVT1, miR-23b-3p or WT1 levels.

### Statistical analysis

Differences were analyzed by a two-tailed Student’s *t*-test or one-way analysis of variance (VANOA), followed by the LSD post hoc test, using SPSS v.22.0 software (SPSS Inc., Chicago, IL, USA). Mann–Whitney U test was used to assess the expression pattern of PVT1, miR-23b-3p and WT1 in the serum of DN patients compared with the normal controls. Statistical significance was determined at *P* < 0.05.

## Results

### PVT1 and WT1 were up-regulated in the serum of DN patients and HG-induced MCs

**Fig. 1:**
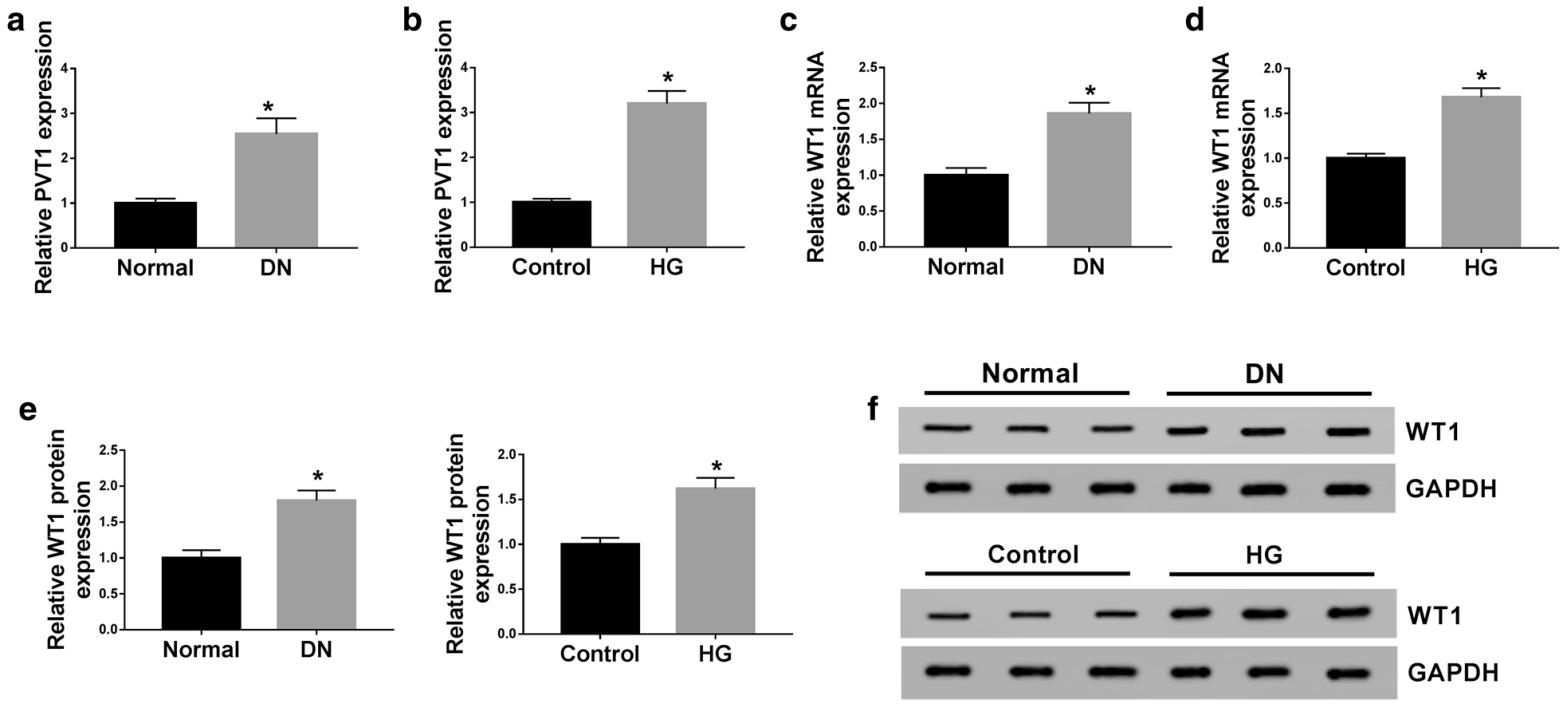
PVT1 and WT1 were up-regulated in the serum of DN patients and HG-induced MCs. PVT1 expression by qRT-PCR in 34 serum samples from patients with DN and 30 serum samples from healthy controls (**a**), HG-induced MCs (**b**). WT1 mRNA level by qRT-PCR in 34 serum samples from patients with DN and 30 serum samples from healthy controls (**c**), HG-induced MCs (**d**). **e**, **f** WT1 expression by western blot in 3 serum samples from DN patients, 3 normal serum samples, normal MCs, and HG-induced MCs. *P < 0.05

Firstly, we determined the expression levels of PVT1 and WT1 in the serum of 34 patients with DN and MCs after HG treatment. As demonstrated by qRT-PCR, PVT1 (Fig. 1a, b) and WT1 mRNA (Fig. 1c, d) levels were significantly increased in the serum of DN patients and HG-induced MCs compared with their counterparts. In line with qRT-PCR, western blot results revealed that compared with a corresponding normal control, WT1 protein level was prominently up-regulated in the serum of DN patients and HG-induced MCs (Fig. 1e, f).

### HG-induced proliferation and fibrosis in MCs were ameliorated by the knockdown of PVT1

**Fig. 2:**
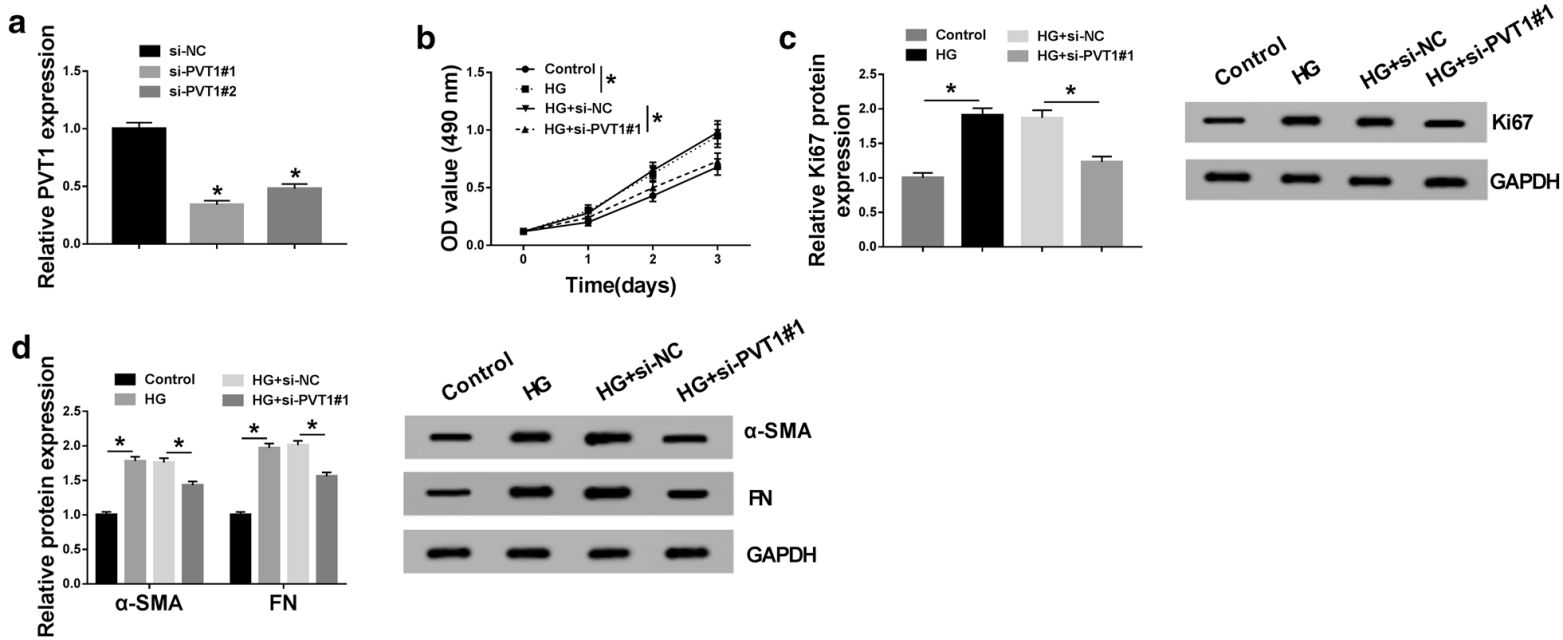
HG-induced proliferation and fibrosis in MCs were alleviated by PVT1 knockdown. **a** PVT1 expression by qRT-PCR in MCs transfected with si-NC, si-PVT1#1, and si-PVT1#2. MCs were exposed to 45 mM of HG or normal medium for 48 h, or transfected with si-NC or si-PVT1#1 before HG treatment, followed by the measurement of cell proliferation by MTS assay 0, 1, 2 and 3 days after transfection (**b**), Ki67 expression by western blot after 48 h transfection (**c**), the levels of α-SMA and FN by western blot 48 h after transfection (**d**). *P < 0.05

To observe the role of PVT1 in DN pathogenesis, loss-of-function experiments were carried out by the transfection of siRNA against PVT1 (si-PVT1#1 and si-PVT1#2). In contrast to the negative group, si-PVT1#1 or si-PVT1#2 transfection triggered a significant reduction in PVT1 expression in MCs (Fig. 2a). Remarkably, si-PVT1#1 induced a more distinct down-regulation in PVT1 level (Fig. 2a). Thus, si-PVT1#1 was selected for further experiments. MTS results showed that compared to the negative control, cell proliferation was strikingly enhanced by HG treatment (Fig. 2b). Western blot analysis revealed that HG treatment led to a distinct increase in proliferation marker Ki67 expression (Fig. 2c), supporting the promotional effect of HG on cell proliferation. Moreover, HG treatment resulted in increased levels of fibrosis-related proteins α-SMA and FN (Fig. 2d), suggesting the enhancement of HG in cell fibrosis. However, HG-induced enhancement in cell proliferation (Fig. 2b, c) and fibrosis (Fig. 2d) was significantly abated by PVT1 depletion when comparing with the negative control.

### HG-induced proliferation and fibrosis in MCs were abated by WT1 silence

**Fig. 3:**
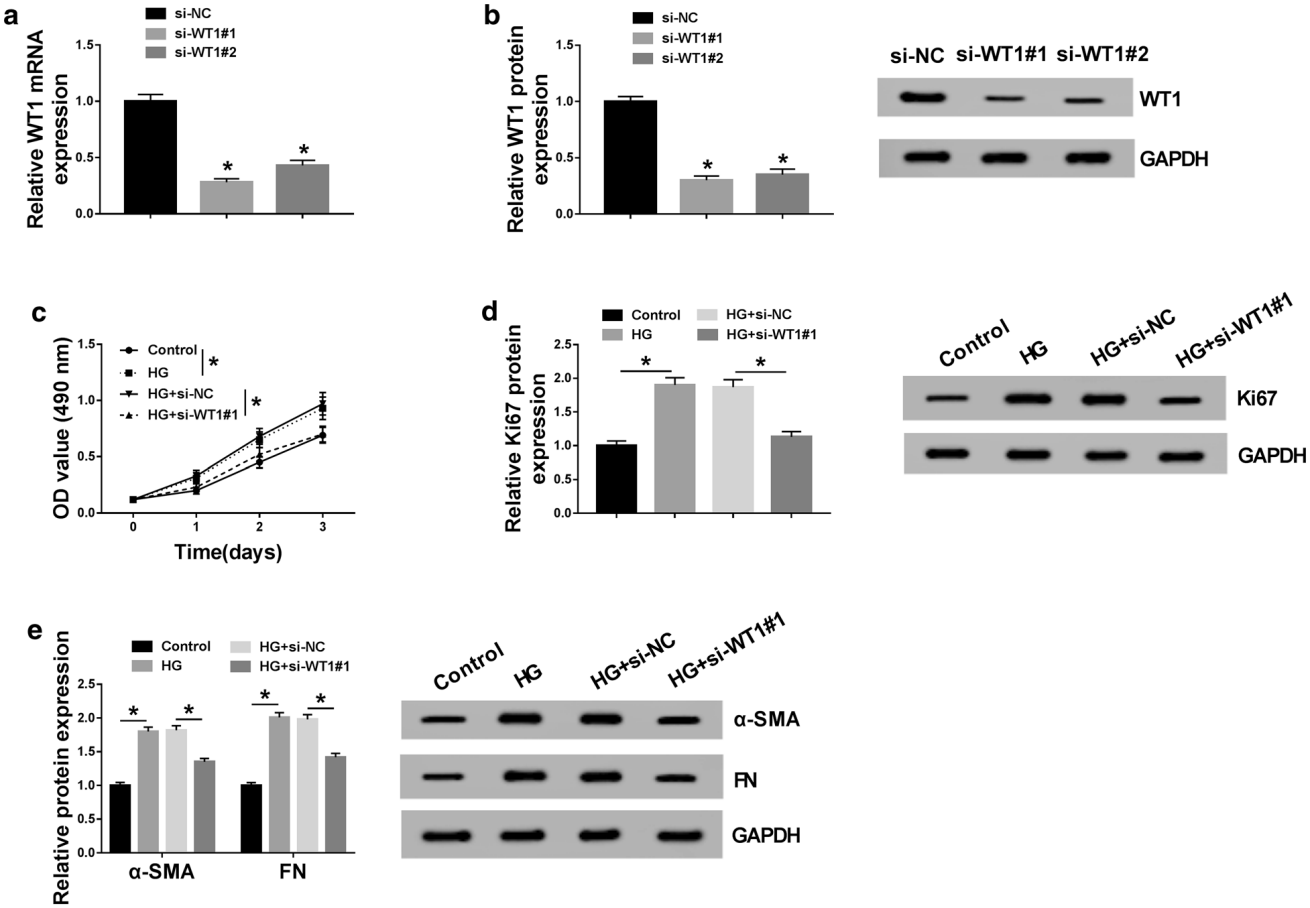
HG-induced proliferation and fibrosis in MCs were abolished by WT1 knockdown. WT1 mRNA expression by qRT-PCR (**a**) and WT1 protein level by western blot (**b**) in MCs transfected with si-NC, si-WT1#1, and si-WT1#2. MCs were treated with 45 mM of HG or normal medium for 48 h, or transfected with si-NC or si-WT1#1 prior to HG exposure, followed by the measurement of cell proliferation by MTS assay 0, 1, 2 and 3 days after transfection (**c**), Ki67 expression by western blot after 48 h transfection (**d**), the levels of α-SMA and FN by western blot 48 h after transfection (**e**). *P < 0.05

To validate the effect of WT1 on DN pathogenesis, we performed “phenocopy” silencing by siRNA against WT1 (si-WT1#1 and si-WT1#2). Transfection of si-WT1#1 or si-WT1#2, but not the si-NC control, markedly decreased WT1 expression at both mRNA and protein levels in MCs (Fig. 3a, b). Notably, si-WT1#1 triggered a more obvious reduction and thus was selected for follow-up experiments (Fig. 3a, b). Subsequently, MCs were transfected with si-WT1#1 or si-NC before HG exposure, followed by the detection of cell proliferation and fibrosis. These results revealed that compared with the negative group, HG-induced proliferation enhancement was strikingly abolished by WT1 depletion (Fig. 3c, d). Moreover, HG-induced fibrosis promotion was highly reversed by the silence of WT1 in MCs (Fig. 3e).

### PVT1 regulated WT1 expression through acting as a miR-23b-3p sponge

**Fig. 4:**
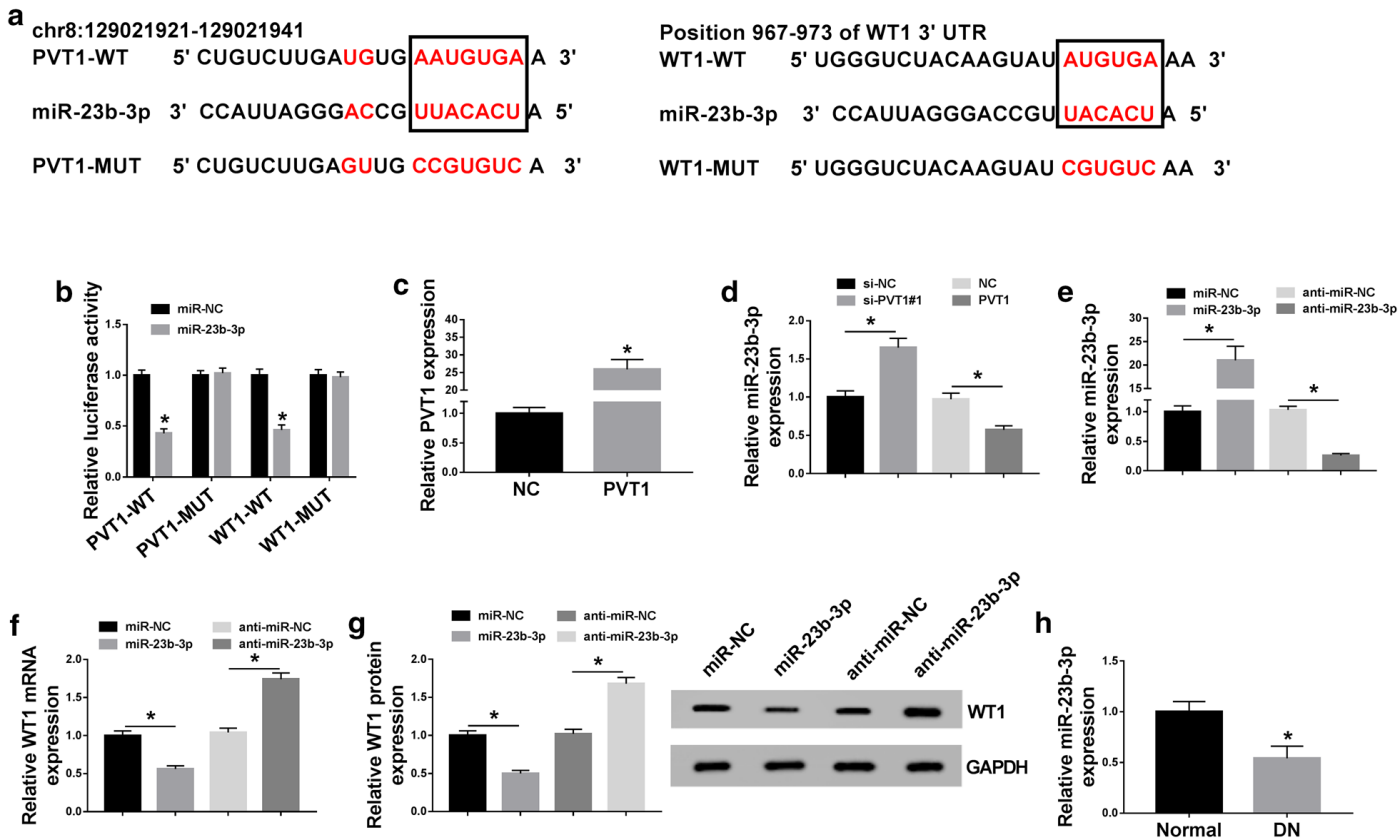
PVT1 acted as a miR-23b-3p sponge and WT1 was a direct target of miR-23b-3p. **a** Schematic of the miR-23b-3p-binding site in PVT1 and WT1 3′-UTR, and mutated miR-23b-3p-binding site. **b** The luciferase activity in MCs transfected with PVT1 wild-type luciferase reporter plasmid (PVT1-WT), WT1 3′-UTR wild-type reporter construct (WT1-WT), or the site-directed mutant of the target sequence (PVT1-MUT and WT1-MUT) together with miR-NC mimic or miR-23b-3p mimic. **c** PVT1 expression by qRT-PCR in MCs transfected with PVT1 overexpression plasmid or the negative plasmid. NC: negative plasmid, PVT1: PVT1 overexpression plasmid. **d** The expression of miR-23b-3p in MCs transfected with si-NC, si-PVT1#1, PVT1 overexpression plasmid, or the negative plasmid. NC: negative plasmid, PVT1: PVT1 overexpression plasmid. MCs were introduced with miR-NC mimic, miR-23b-3p mimic, anti-miR-NC or anti-miR-23b-3p, followed by the determination of miR-23b-3p expression by qRT-PCR (E), WT1 mRNA expression by qRT-PCR (**f**), WT1 protein level by western blot (**g**). **h** MiR-23b-3p expression by qRT-PCR in 34 serum samples from patients with DN and 30 serum samples from healthy controls. *P < 0.05

To explore the underlying mechanism by which PVT1 regulated HG-induced proliferation and fibrosis in MCs, the online software miRcode was used to help identify the miRNAs possessing a potential to interact with PVT1. These data revealed that PVT1 harbored a putative target sequence of miR-23b-3p (Fig. 4a). To validate this, dual-luciferase reporter assays were implemented by PVT1 wild-type luciferase reporter plasmid (PVT1-WT) containing the miR-23b-3p-binding sites and its mutant in the seed region (PVT1-MUT). Transient introduction of miR-23b-3p mimic, but not a scrambled control sequence, significantly inhibited the luciferase activity of PVT1-WT (Fig. 4b). However, the site-directed mutant of the miR-23b-3p-binding sites remarkably abolished the effect of miR-23b-3p on reporter gene expression (Fig. 4b). Additionally, RNA pulldown results revealed that the enrichment of PVT1 was significantly increased by bio-miR-23b-3p-WT, and this effect was abrogated by bio-miR-23b-3p-MUT (Additional file 1: Fig. S1A). Then, we observed whether the miR-23b-3p-binding sites were functional. qRT-PCR data showed that PVT1 expression was highly elevated by the transfection of PVT1 overexpression plasmid compared with the negative plasmid (Fig. 4c). As expected, miR-23b-3p expression was significantly increased by PVT1 knockdown, while it was markedly decreased when PVT1 overexpression (Fig. 4d). These data together established a notion that PVT1 acted as a molecular sponge of miR-23b-3p.

It is widely acknowledged that miRNAs exert biological function through the posttranscriptional repression of their target mRNAs [20]. Thus, to understand the role of miR-23b-3p, we carried out a detailed analysis for its molecular targets. Using the online software TargetScan Human 7.1, the data revealed a potential complementary sequence of miR-23b-3p in WT1 3′-UTR (Fig. 4a). Transfection of WT1 3′-UTR wild-type reporter construct in the presence of miR-23b-3p mimic induced a significant reduction of relative luciferase activity, and this effect was strikingly abrogated by the mutant of the target sequence (Fig. 4b). The data of RIP assays showed that compared with the negative control, miR-23b-3p and WT1 were simultaneously enriched by anti-Ago2 antibody (Additional file 1: Fig. S1B). As shown by qRT-PCR, miR-23b-3p expression was highly increased by miR-23b-3p mimic transfection and decreased after the introduction of anti-miR-23b-3p in MCs (Fig. 4e). Moreover, in comparison to their counterparts, the mRNA and protein levels of WT1 were significantly reduced by miR-23b-3p overexpression, while they were prominently elevated when miR-23b-3p depletion (Fig. 4f, g). Additionally, the data of qRT-PCR showed that miR-23b-3p was down-regulated in the serum of DN patients (Fig. 4h). These data together indicated that WT1 was directly targeted and repressed by miR-23b-3p.

**Fig. 5:**
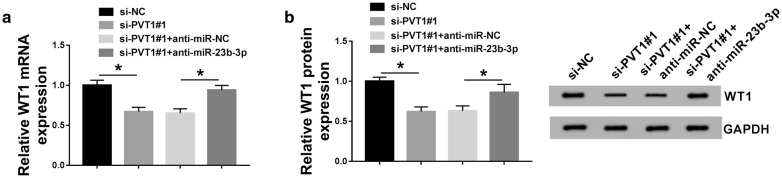
PVT1 regulated WT1 expression through acting as a ceRNA of miR-23b-3p. MCs were transfected with si-NC, si-PVT1#1, si-PVT1#1 + anti-miR-NC, or si-PVT1#1 + anti-miR-23b-3p, followed by the detection of WT1 mRNA expression by qRT-PCR (**a**), WT1 protein level by western blot (**b**). *P < 0.05

LncRNAs are widely accepted to modulate gene expression through acting as ceRNAs of miRNAs [6]. We further detected whether PVT1 regulated WT1 expression in MCs. As expected, WT1 mRNA and protein levels were remarkably suppressed by PVT1 knockdown compared with the negative group (Fig. 5a, b). Nevertheless, these effects were prominently abolished by the cotransfection of anti-miR-23b-3p (Fig. 5a, b). Taken together, these results strongly pointed out the role of PVT1 as a molecular sponge of miR-23b-3p to protect against WT1 repression.

### The ameliorated effect of PVT1 knockdown on HG-induced proliferation and fibrosis in MCs was mediated by the miR-23b-3p/WT1 axis

**Fig. 6:**
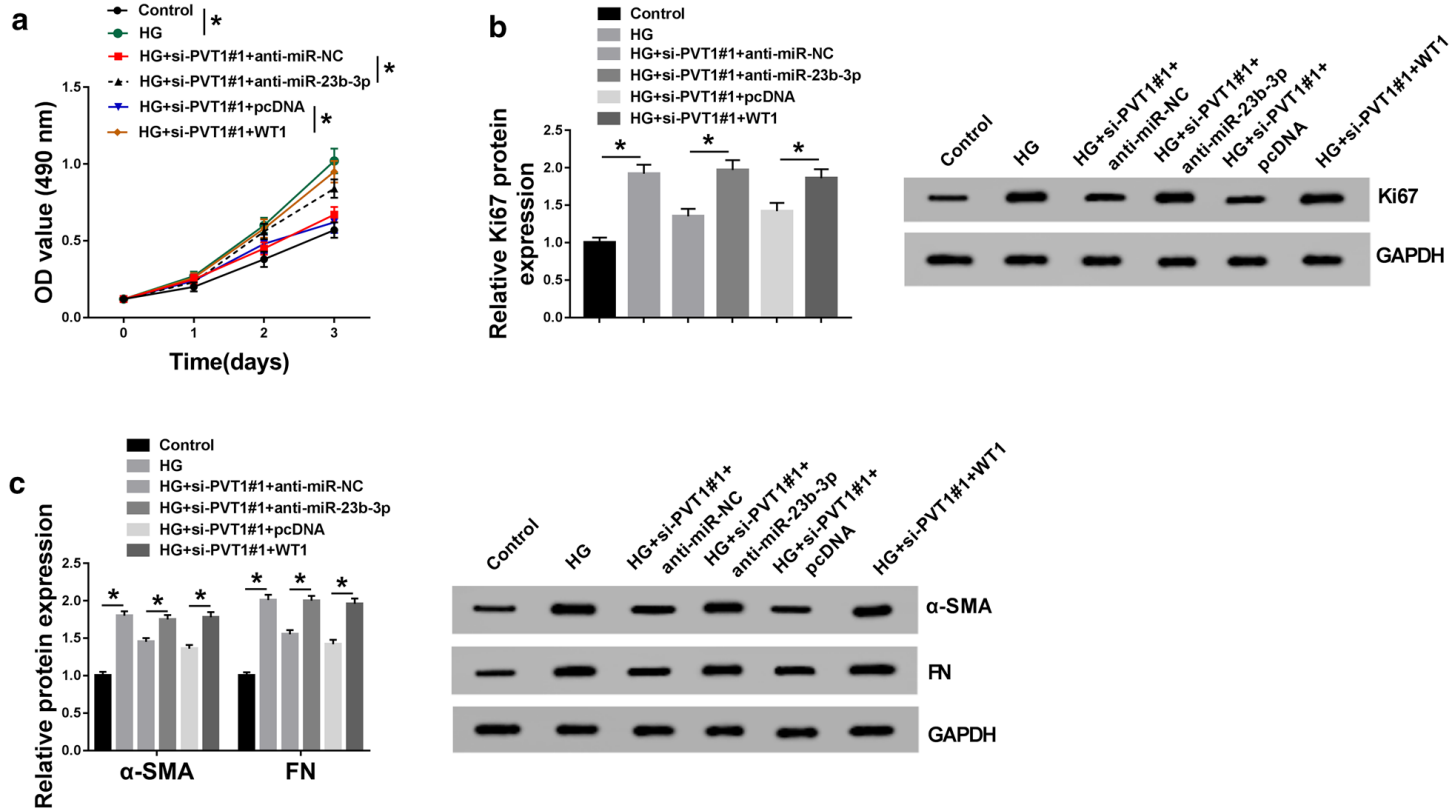
PVT1 knockdown ameliorated HG-induced proliferation and fibrosis by miR-23b-3p/WT1 axis. MCs were transfected with si-PVT1#1 + anti-miR-NC, si-PVT1#1 + anti-miR-23b-3p, si-PVT1#1 + pcDNA, or si-PVT1#1 + WT1 overexpression plasmid before HG treatment, followed by the determination of cell proliferation by MTS assay (**a**), Ki67 expression by western blot (**b**), the levels of α-SMA and FN by western blot (**c**). pcDNA: nontarget negative plasmid, WT1: WT1 overexpression plasmid. *P < 0.05

To provide further mechanistic insight into the link between PVT1 and miR-23b-3p/WT1 axis on HG-induced MCs, the cells were cotransfected with si-PVT1#1 and anti-miR-23b-3p or WT1 overexpression plasmid. As a result, in contrast to their counterparts, si-PVT1#1-mediated anti-proliferation (Fig. 6a, b) and anti-fibrosis (Fig. 6c) effects were significantly abolished by the cotransfection of anti-miR-23b-3p or WT1 overexpression plasmid. These data together suggested that PVT1 knockdown might ameliorate HG-induced MCs proliferation and fibrosis by the miR-23b-3p/WT1 axis.

### NF-κB signaling pathway was involved in the regulation of the PVT1/miR-23b-3p/WT1 axis in HG-induced MCs

**Fig. 7:**
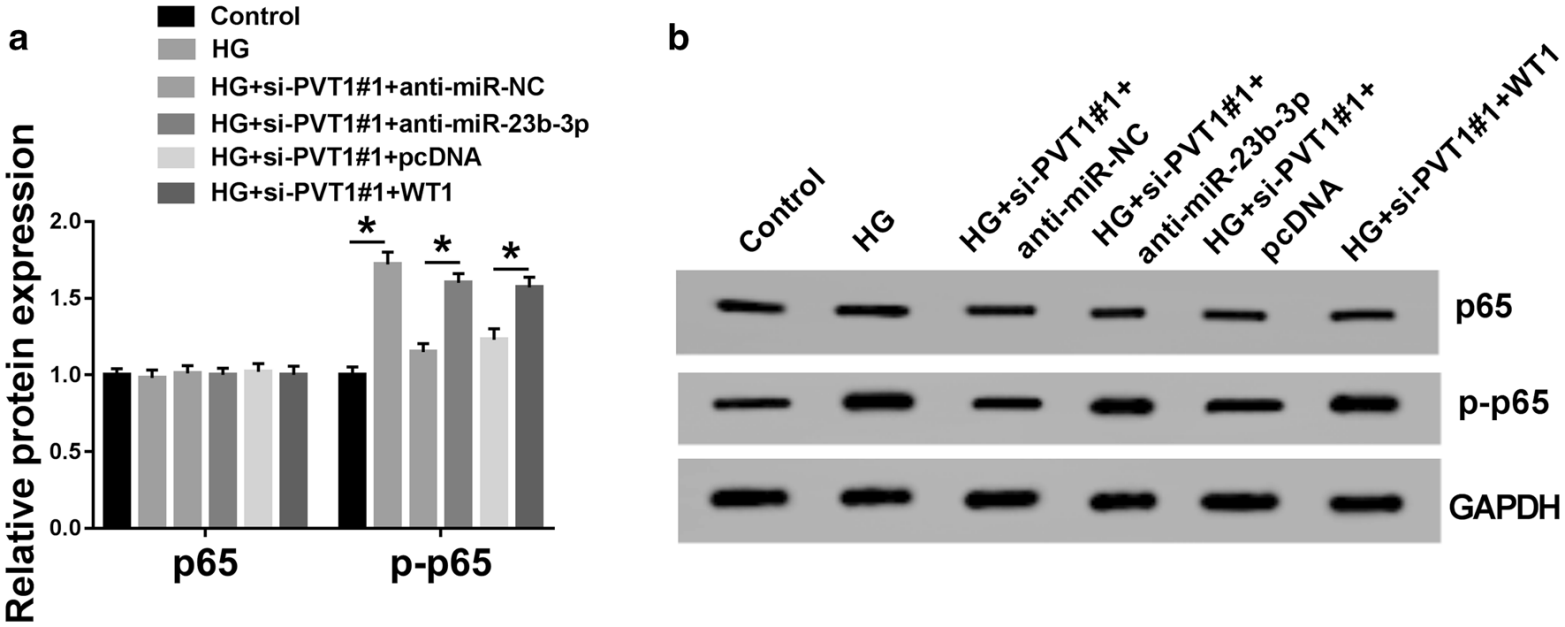
NF-κB signaling pathway was involved in PVT1-mediated regulation in HG-induced MCs via miR-23b-3p/WT1 axis. **a**, **b** The levels of p65 and p-p65 by western blot in MCs treated with HG or normal medium, or transfected with si-PVT1#1 + anti-miR-NC, si-PVT1#1 + anti-miR-23b-3p, si-PVT1#1 + pcDNA, or si-PVT1#1 + WT1 overexpression plasmid before HG treatment. pcDNA: nontarget negative plasmid, WT1: WT1 overexpression plasmid. *P < 0.05

NF-κB signaling, an important inflammation-related pathway, is implicated in the development and progression of DN [21]. Therefore, we further investigated whether PVT1 could regulate this signaling pathway in HG-induced MCs. As demonstrated by western blot, p-p65 level was up-regulated in HG-induced MCs compared with control group (Fig. 7a, b). These data also showed that the knockdown of PVT1 mitigated the activation of the NF-κB signaling, as evidenced by the decrease in p-p65 level (Fig. 7a, b). More interestingly, in comparison to a corresponding negative group, the decreased effect of PVT1 depletion on p-p65 expression was significantly abolished by the cotransfection of anti-miR-23b-3p or WT1 overexpression plasmid (Fig. 7a, b). Together, these data suggested that PVT1 knockdown might relieve HG-induced proliferation and fibrosis in MCs by blocking the NF-κB signaling pathway via miR-23b-3p/WT1 axis.

## Discussion

**Fig. 8:**
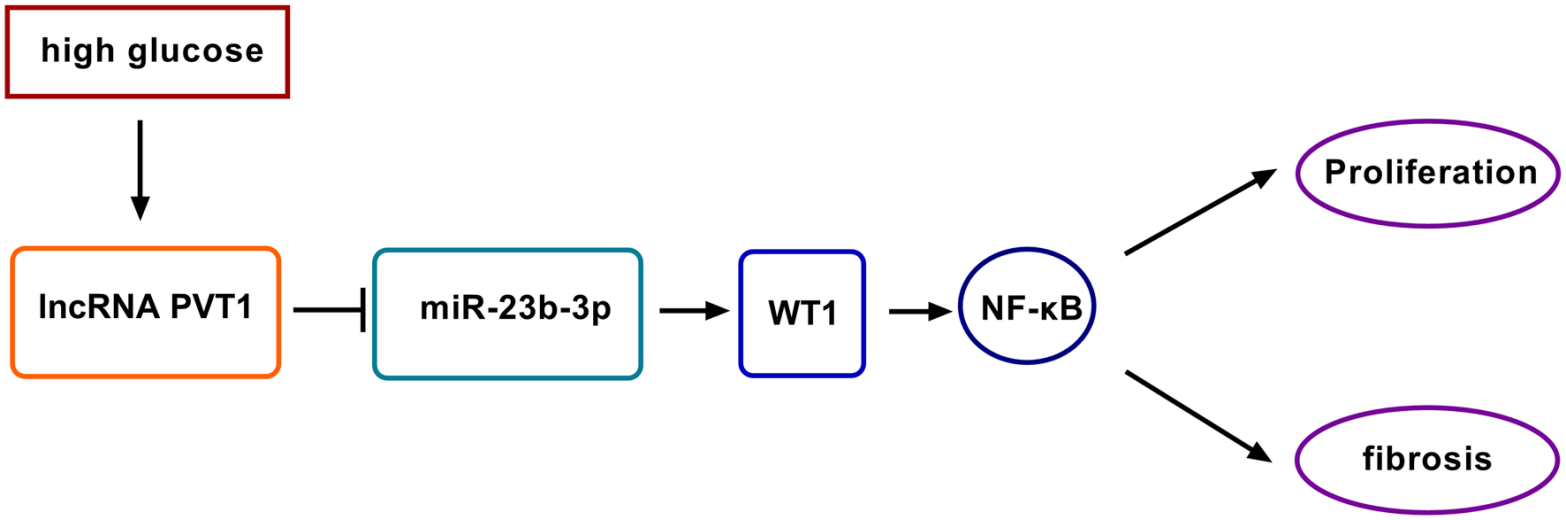
Schematic model of the PVT1/miR-23b-3p/WT1/NF-κB pathway axis in HG-induced proliferation and fibrosis in MCs. HG treatment induced PVT1 expression in MCs. Then, the elevated expression of PVT1 repressed miR-23-3p level and thus protected against WT1 repression. Next, the NF-κB signaling pathway was activated. Finally, the activation of the NF-κB signaling pathway enhanced cell proliferation and fibrosis of HG-induced MCs, which accelerated DN progression

Emerging evidence has recently suggested that lncRNAs are essential regulators in the hyperglycemia and pathogenesis of DN, offering a possibility of lncRNAs as potential biomarkers for DN development and progression [22–24]. In this study, we were first to uncover that the knockdown of PVT1 ameliorated HG-induced proliferation and fibrosis in human MCs partially through targeting the miR-23b-3p/WT1/NF-κB pathway (Fig. 8).

PVT1 has been identified to be up-regulated in many tumor tissues and it contributes to the pathophysiology of human cancers [25, 26]. In the present study, our results demonstrated that PVT1 was up-regulated in the serum of DN patients and HG-induced MCs, consistent with a previous study [12]. Previous researches had manifested that PVT1 might mediate DN pathogenesis via mechanisms including the accumulation of ECM [12, 27]. To further investigate the function of PVT1 in DN progression, we performed loss-of-function experiments in HG-induced MCs, and our results indicated that PVT1 depletion attenuated HG-induced proliferation and fibrosis in human MCs. In short, the knockdown of PVT1 repressed DN progression possibly through modulating MCs proliferation and fibrosis. Similar to our findings, Li et al. reported that lncRNA LINC00968 was high-expressed in HG-induced MCs, and its depletion mitigated HG-induced proliferation and fibrosis in MCs through the repression of p21 expression [9]. Gao et al. manifested that lncRNA-NR 033515 enhanced the proliferation and fibrosis in HG-induced mouse MCs through sponging miR-743b-5p [28].

WT1, a transcription factor and regulator of podocyte differentiation and homeostasis, is aberrantly expressed in various types of human cancers, such as uterine cancer and acute myeloid leukemia [29, 30]. WT1 was also reported to implicate in focal segmental glomerulosclerosis via mediating miR-193a [31]. In this study, our data firstly validated that WT1 was up-regulated in the serum of DN patients and HG-induced MCs. Moreover, we firstly demonstrated that WT1 silencing relieved HG-induced proliferation and fibrosis in human MCs.

It is widely accepted that lncRNAs protect against gene expression repression via acting as sponges of miRNAs, and consequently preventing specific miRNAs from binding to their target mRNAs [6]. Thus, the online software miRcode was used to help identify the miRNAs possessing a potential to interact with PVT1. Among these predicted candidates, miR-23b-3p was of particular interest because miR-23b expression had been found to be down-regulated in the peripheral blood from diabetes patients and the kidneys of diabetes animal model [32, 33]. Zhao et al. also reported that HG reduced miR-23b expression in HK-2 cells, and high level of miR-23b ameliorated fibrosis and albuminuria in DN [32]. Liu et al. underscored that miR-23b up-regulation mitigated HG-induced epithelial-to-mesenchymal transition in HK-2 cells [33]. Additionally, using the TargetScan Human 7.1 software, the data revealed a potential complementary sequence of miR-23b-3p in WT1 3′-UTR. Subsequently, we firstly confirmed that PVT1 sequestered miR-23b-3p through acting as a miR-23b-3p sponge and WT1 was directly targeted and suppressed by miR-23b-3p using dual-luciferase reporter, RIP, qRT-PCR and western blot assays. More intriguingly, we were first to validate that PVT1 modulated WT1 expression through sponging miR-23b-3p. Moreover, the miR-23b-3p/WT1 axis mediated the protective role of PVT1 knockdown on HG-induced proliferation and fibrosis in MCs.

NF-κB signaling pathway has been postulated to be involved in the development and pathogenesis of DN [34, 35]. Moreover, HG promoted MCs proliferation and monocyte chemoattractant protein-1 expression partially through the NF-κB pathway [36]. In the present study, our data substantiated that the NF-κB signaling pathway was involved in the regulation of the PVT1/miR-23b-3p/WT1 axis in HG-induced MCs. Feng et al. demonstrated that PVT1 modulated inflammatory response and cardiac function in a sepsis model through regulating the NF-κB signaling pathway [37]. Naidu et al. discovered that miR-23b mediated the regulation in lung tumorigenesis via targeting the NF-κB signaling pathway [38]. This study was limited in vitro investigation, and more in vivo researches about the novel mechanism using the DN animal model will be conducted in further work.

Additionally, when we observed the impact of HG treatment on cell proliferation and fibrosis, we used the same concentration of d-mannitol (HM) to treat the MCs. Our data demonstrated that cell proliferation, fibrosis, and Ki67 and p-p65 levels were not significantly affect by HM treatment (Additional file 2: Fig. S2). Therefore, these experiments excluded the possibility that the effect of HG on MCs could be due to the changes in the osmolarity of the culture medium.

## Conclusion

In conclusion, our study suggested that the knockdown of PVT1 ameliorated HG-induced MCs proliferation and fibrosis at least partially by targeting the miR-23b-3p/WT1/NF-κB pathway. Thus, lncRNAs might provide novel potential therapeutic agents for DN treatment.

## Supplementary information

**Additional file 1: Fig. S1.** The correlation between PVT1 and miR-23b-3p or WT1 was detected using the RNA pulldown or RIP assays. (A) RNA pulldown assay for the correlation between PVT1 and miR-23b-3p using bio-NC, bio-miR-23b-3p-WT or bio-miR-23b-3p-MUT. (B) RIP assays for the correlation between miR-23b-3p and WT1 using anti-Ago2 or anti-IgG antibody. *P < 0.05.

**Additional file 2: Fig. S2.** The impact of D-mannitol (HM) on MCs proliferation, fibrosis and p-p65 level. MCs were treated with HM for 48 h, followed by the measurement of cell proliferation (A), Ki-67 level (B), the levels of α-SMA and FN (C), p65 and p-p65 levels (D) by western blot. *P < 0.05 or n.s. meant no significant difference.

## Data Availability

The analyzed data sets generated during the present study are available from the corresponding author on reasonable request.
