# Supplementary figures and images for: Knockdown of lncRNA PVT1 alleviates high glucose-induced proliferation and fibrosis in human mesangial cells by miR-23b-3p/WT1 axis
Source: Diabetol Metab Syndr. 2020 Apr 15;12:33. doi: 10.1186/s13098-020-00539-x (PMC7161221; doi:10.1186/s13098-020-00539-x)

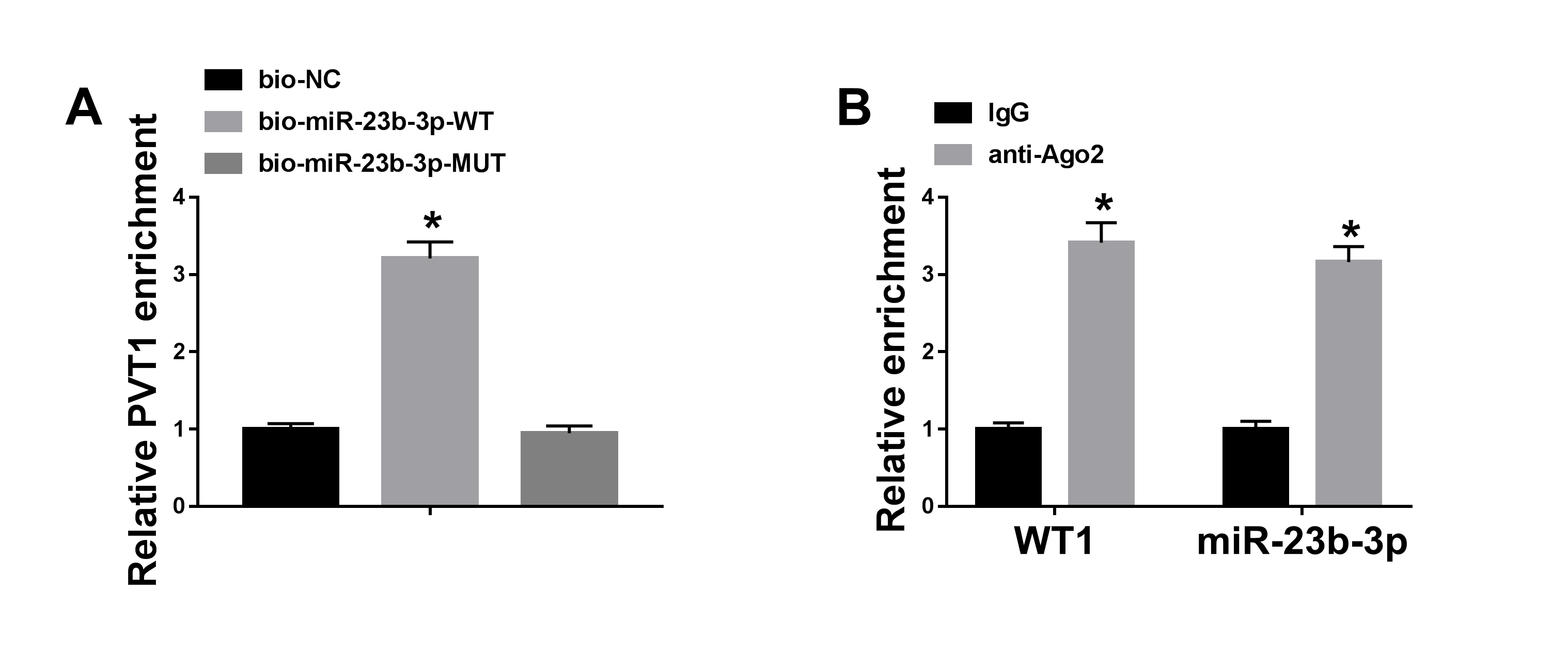

Supplement: Supplementary file 1 — Additional file 1: Fig. S1. The correlation between PVT1 and miR-23b-3p or WT1 was detected using the RNA pulldown or RIP assays. (A) RNA pulldown assay for the correlation between PVT1 and miR-23b-3p using bio-NC, bio-miR-23b-3p-WT or bio-miR-23b-3p-MUT. (B) RIP assays for the correlation between miR-23b-3p and WT1 using anti-Ago2 or anti-IgG antibody. *P < 0.05. [file 13098_2020_539_MOESM1_ESM.tif]

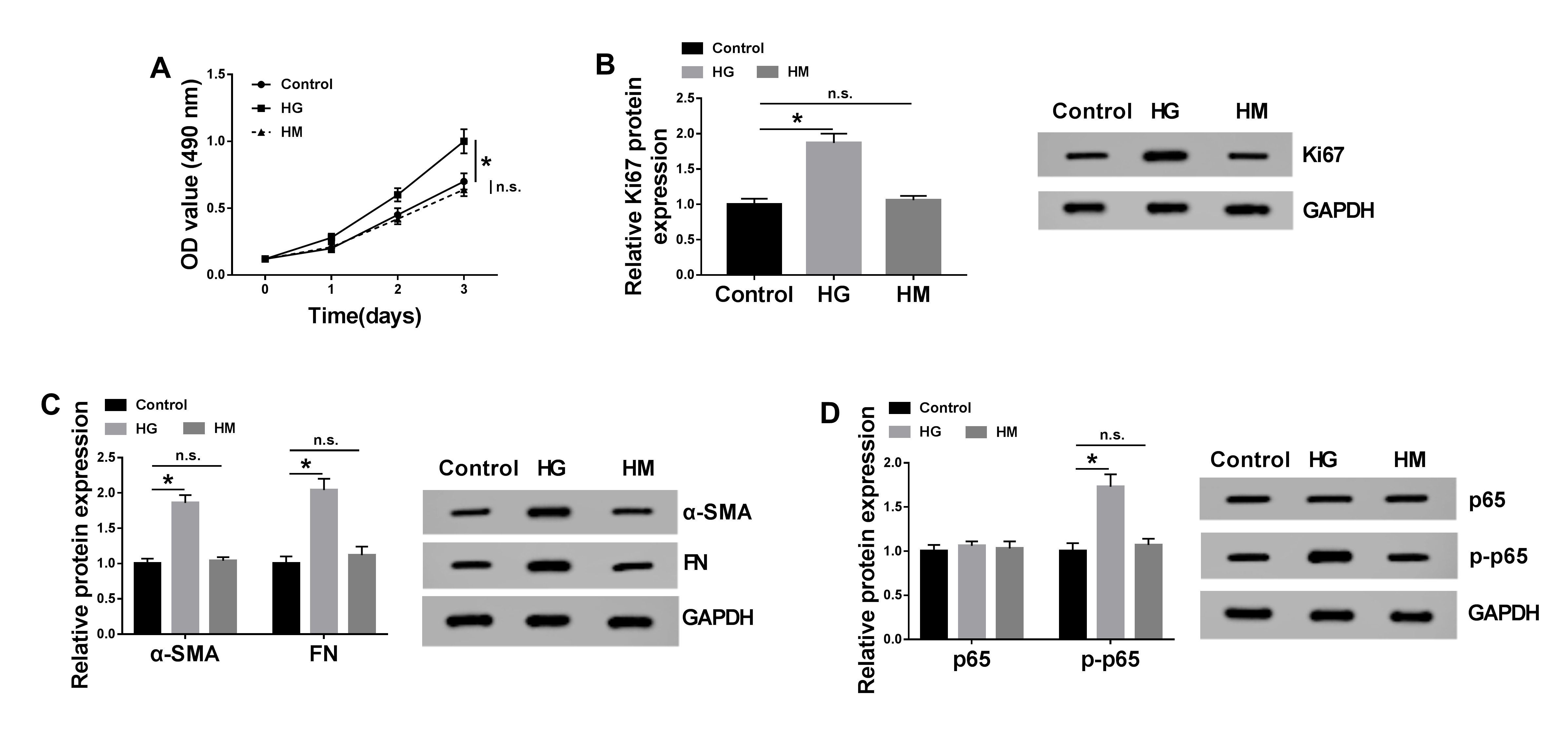

Supplement: Supplementary file 2 — Additional file 2: Fig. S2. The impact of D-mannitol (HM) on MCs proliferation, fibrosis and p-p65 level. MCs were treated with HM for 48 h, followed by the measurement of cell proliferation (A), Ki-67 level (B), the levels of α-SMA and FN (C), p65 and p-p65 levels (D) by western blot. *P < 0.05 or n.s. meant no significant difference. [file 13098_2020_539_MOESM2_ESM.tif]
